# Mosaicism in Fanconi anemia: concise review and evaluation of published cases with focus on clinical course of blood count normalization

**DOI:** 10.1007/s00277-020-03954-2

**Published:** 2020-02-17

**Authors:** Eileen Nicoletti, Gayatri Rao, Juan A. Bueren, Paula Río, Susana Navarro, Jordi Surrallés, Grace Choi, Jonathan D. Schwartz

**Affiliations:** 1 Rocket Pharmaceuticals, Inc., New York, NY USA; 2 Hematopoietic Innovative Therapies Division, Centro de Investigaciones Energéticas, Medioambientales y Tecnológicas (CIEMAT), Madrid, Spain; 3 Centro de Investigación Biomédica en Red de Enfermedades Raras (CIBERER), Madrid, Spain; 4 Instituto de Investigaciones Sanitarias Fundación Jiménez Díaz (IIS-FJD), Madrid, Spain; 5 Genome Instability and DNA Repair Syndromes Group, Department of Genetics and Microbiology, Universitat Autònoma de Barcelona (UAB), Barcelona, Spain; 6 Servicio de Genética e Instituto de Investigaciones Biomédicas del Hospital de Sant Pau, Barcelona, Spain

**Keywords:** Fanconi anemia, Bone marrow failure, Autologous stem cell transplantation, Gene therapy, Mosaicism

## Abstract

Fanconi anemia (FA) is a DNA repair disorder resulting from mutations in genes encoding for FA DNA repair complex components and is characterized by variable congenital abnormalities, bone marrow failure (BMF), and high incidences of malignancies. FA mosaicism arises from reversion or other compensatory mutations in hematopoietic cells and may be associated with BMF reversal and decreased blood cell sensitivity to DNA-damaging agents (clastogens); this sensitivity is a phenotypic and diagnostic hallmark of FA. Uncertainty regarding the clinical significance of FA mosaicism persists; in some cases, patients have survived multiple decades without BMF or hematologic malignancy, and in others hematologic failure occurred despite the presence of clastogen-resistant cell populations. Assessment of mosaicism is further complicated because clinical evaluation is frequently based on clastogen resistance in lymphocytes, which may arise from reversion events both in lymphoid-specific lineages and in more pluripotent hematopoietic stem/progenitor cells (HSPCs). In this review, we describe diagnostic methods and outcomes in published mosaicism series, including the substantial intervals (1–6 years) over which blood counts normalized, and the relatively favorable clinical course in cases where clastogen resistance was demonstrated in bone marrow progenitors. We also analyzed published FA mosaic cases with emphasis on long-term clinical outcomes when blood count normalization was identified. Blood count normalization in FA mosaicism likely arises from reversion events in long-term primitive HSPCs and is associated with low incidences of BMF or hematologic malignancy. These observations have ramifications for current investigational therapeutic programs in FA intended to enable gene correction in long-term repopulating HSPCs.

**Electronic supplementary material:**

The online version of this article (10.1007/s00277-020-03954-2) contains supplementary material, which is available to authorized users.

## Fanconi anemia and mosaicism

### Fanconi anemia overview

Fanconi anemia (FA) is a deoxyribonucleic acid (DNA) repair disorder resulting from mutations in genes encoding for protein components of the FA/BRCA DNA repair pathway. More than 20 identified proteins interact as components of the FA core, FANCI, FANCD2 and downstream complexes. In conjunction with other DNA repair elements, FA proteins facilitate repair of interstrand crosslinks (ICLs) and other forms of DNA damage [1, 2]. FA proteins also contribute to other cellular processes beyond DNA repair, including cellular responses to oxidative stress and inflammatory stimuli [3–5].

FA is characterized by frequent bone marrow failure (BMF), myeloid malignancies, and epithelial cancers [1, 6]. The BMF and neoplastic manifestations of FA are frequently but not universally accompanied by congenital abnormalities including growth retardation/short stature, microcephaly, and abnormalities of skin pigmentation, upper limbs (especially thumbs and radii), gonads, kidneys, and other organ systems. Manifestations of BMF are frequently noted during the first decade of life; the incidences of myeloid and solid organ malignancies increase during the second and third decades of life, respectively, such that the cumulative incidence of acute myeloid leukemia (AML)/myelodysplastic syndrome (MDS) is approximately 30% by age 30 and the cumulative incidence of solid organ cancers approaches 40% by age 40 [6, 7].

### Fanconi anemia mosaicism

Somatic mosaicism in FA (hereafter referred to as mosaicism) arises from reversion or other compensatory mutations in hematopoietic stem cell/progenitor cells (HSPCs) from which arise a population of bone marrow and blood cells with a functional DNA repair capacity. Patients in whom a reversion in a pathogenic FA mutation has occurred frequently have two distinct blood cell populations. One population is sensitive to DNA-damaging agents (mitomycin-C [MMC] or diepoxybutane [DEB]) and consistent with an FA diagnosis and another population resistant to these DNA-damaging (or clastogenic) agents – hence the “mosaic” label indicating the coexistence of phenotypically distinct cellular components [8–10]. The purpose of this review is to summarize the existing literature regarding FA mosaicism, with particular emphasis on persistent diagnostic and prognostic uncertainties. We provide an evaluation of case series in which FA mosaic patients were followed clinically over years (and at times decades), with emphasis on the clinicopathologic relevance of laboratory diagnostic evaluations of these cases. Additionally, we conducted a literature-based assessment of FA mosaic cases most likely emanating from reversion or other compensatory mutations in long-term repopulating HSC populations.

**Fig. 1:**
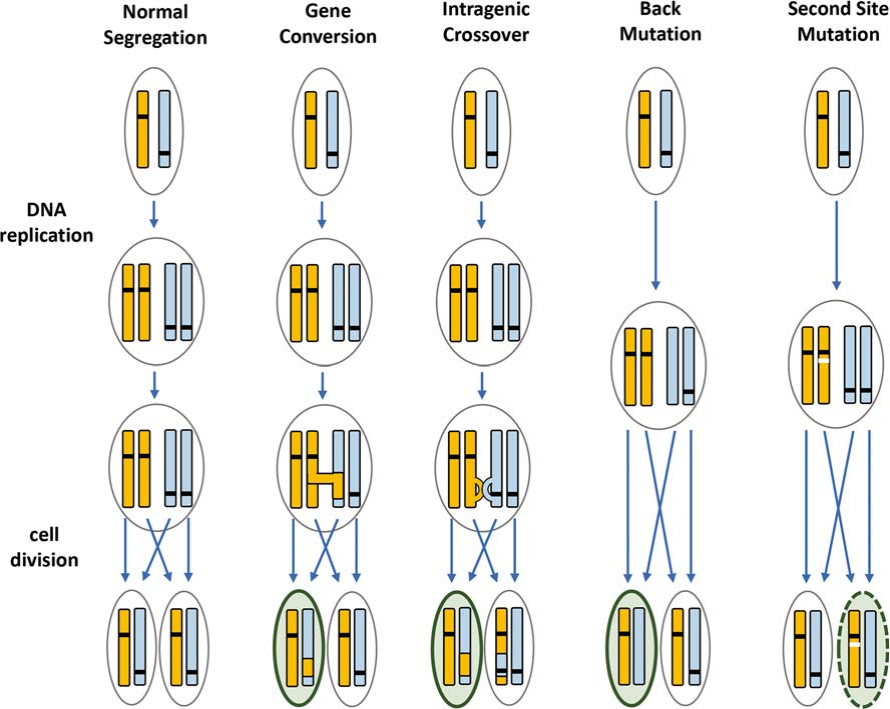
Several mechanisms by which additional chromosomal rearrangements or mutations may facilitate correction of an inherited recessive genetic disorder. The yellow and blue bars indicate an individual gene, with black hashes indicating a disease-causing mutation. Reversion mutations may arise either during or subsequent to DNA replication and may involve transfer of genetic material between paired chromosomes (gene conversion or intragenic crossover) or mutations within a single chromosome (and gene). Gene conversion, intragenic crossover, and back mutations result in genetic correction in one allele within a daughter cell (indicated by the green border and background). Second-site mutations (white hash) result in a gene capable of generating a functional protein (indicated by the green dashed border and green background) in one allele, although the gene itself may differ from wildtype. Adapted from Pasmooij et al. [17]

Mosaicism in FA patients was noted prior to initial identification of the first *FANC* gene (*FANCC*) in 1992 [11], with initial reports describing FA patients in whom 60–80% of cultured lymphocytes (either PHA-stimulated T cells or EBV-transformed B cells) displayed resistance to concentrations of alkylating agents typically toxic to FA patient cells [12, 13]. Several mechanisms by which an additional genetic event may result in a functional FA gene and protein have been identified and include gene conversion, intragenic crossover, back mutation, and second-site mutation – each of which may result in a restoration of the affected gene to wildtype [8, 14, 15]. Second-site mutations involve either a compensatory point mutation (insertion or deletion) at a distinct site within a mutated gene, resulting in a functional gene and protein that may be nonetheless distinct from wildtype [15–17]. The mechanisms by which these various mutations may result in cell populations expressing a functional gene and/or protein are illustrated in Fig. 1.

### Mosaicism in other genetic disorders

Reversion mutations and resultant mosaicism have been reported in Bloom syndrome, X-linked and adenosine deaminase severe combined immunodeficiency (X-SCID and ADA-SCID), Diamond Blackfan anemia (DBA), Wiskott-Aldrich syndrome, epidermolysis bullosa, tyrosinemia, and dyskeratosis congenita (DC) [18–25]. The extent of phenotypic correction in any clinical setting is dependent on both the chronology of a reversion event either during embryonic or postnatal development and the degree by which a disorder manifests in hierarchical (as opposed to heterarchical) organ systems. Disorders predominantly affecting blood cells may be particularly reversible because of the hierarchical nature of hematopoiesis, with partial or comprehensive phenotypic corrections identified most frequently in the context of normalization within a single lineage [19–21]. Multilineage mosaicism has been reported in DC [25]. In several of these conditions and in FA in particular, the underlying disorder predisposes to heightened genetic instability, enhancing the potential for additional mutations, many of which are potentially deleterious (resulting in characteristic frequent malignancy in Bloom syndrome, DBA, DC, and FA), but in more limited instances may confer a beneficial phenotypic reversion. A particularly unstable intragenic duplication was recently characterized in a FANCB patient with mosaicism associated with mild hematologic deficits. The instability of this duplication was evident not only from a decreasing percentage of aberrant blood cells over 11 years of observation but from the presence of a modest population (8%) of fibroblasts displaying a wild-type allele, representing the first identification of reversion in a non-hematopoietic cell population in FA [26].

### FA mosaicism: selective growth advantage and prognostic uncertainty

In the abovementioned diseases, mosaicism has been described as “natural gene therapy” [27, 28], and a selective growth advantage of revertant cells in selected disorders and organ systems (including the hematologic component of FA) has long been hypothesized. The potential for a marked proliferative advantage at the HSC level has been most profoundly illustrated by a case of monozygotic twin sisters diagnosed with FA early in life who nonetheless had demonstrated hematologic stability during 28 years of reported follow-up, with clastogen resistance evident in peripheral blood lymphocytes despite characteristic fragility of cultured fibroblasts. Analysis of bone marrow progenitors indicated multilineage presence of the compensatory mutation, consistent with a hypothesized prenatal reversion in a long-term HSC, resulting in multilineage engraftment via shared in utero circulation [28, 29]. This example of gene reversion and selective growth advantage enabling comprehensive and sustained hematopoiesis has represented a naturally occurring proof of concept for the potential hematologic correction of FA via gene therapy. A selective advantage of gene-corrected FA cells has been demonstrated in murine FA models [30–32], human embryonic stem cells [33], induced pluripotent stem cells [34], and gene-corrected cells from FA patients transplanted in immunocompromised mice [35]; these observed in vivo selective advantages have been largely facilitated by administration of cytotoxic conditioning agents. A selective advantage has also recently been demonstrated in nonconditioned FA-A patients receiving gene-corrected autologous CD34+ cells, by means of progressive increases in gene-corrected and clastogen-resistant lymphocytes and marrow progenitors during 18–30 months of follow-up [36].

The presence of a revertant FA hematopoietic cell population has often been associated with a favorable hematologic course in FA patients. However, the presence of clastogen-resistant blood and marrow cells may not uniformly be associated with long-term BMF- or AML-free survival. Gregory and colleagues reported a male patient with hematologic stability during the initial 9–17 years of life and a stable population of DEB-resistant peripheral blood lymphocytes (61–74%). Analysis of bone marrow progenitors at age 15 indicated multilineage presence of the revertant mutation (including lymphoid, myeloid, and erythroid progenitors). At ages 15 and 17, this patient nonetheless developed evidence of an 11q deletion in a population of non-revertant bone marrow cells – a cytogenetic abnormality potentially associated with myeloid malignancy [37]. The prognostic uncertainty regarding the presence of a hematopoietic population containing a reversion mutation is rendered additionally complex because the diagnostic methods utilized in FA are most frequently directed at a differentiated T-lymphoid cell population that may be genetically divergent from multilineage stem and progenitor populations. The diagnosis and clinical prognosis of FA mosaic patients during recent decades are detailed subsequently.

### Mosaicism diagnosis

Diagnosis of mosaicism in FA is contingent on demonstration of a population of blood and/or bone marrow cells that display resistance to concentrations of DNA-damaging agents that are typically toxic to FA hematopoietic cells. Diagnostic challenges persist because mosaicism may be restricted to specific cell lineages and evaluation of multiple blood and marrow lineages is frequently not clinically feasible. The percentage of resistant cells within a single lineage may not be indicative of the overall hematologic milieu and (especially at a single timepoint) may not provide clinically meaningful prognostic information.

A critical component of an FA diagnosis and of potential FA mosaicism involves evaluation of cultured T lymphocytes in the presence of DNA-damaging agents, most frequently DEB or MMC. Lymphocytes from FA patients frequently display marked chromosomal instability (also referred to as fragility, evidenced by chromosomal breaks and radial figures) in the presence of clastogen concentrations that result in minimal abnormalities in non-FA cells [9, 10]. Some centers, including those involved in the evaluation of a large and rigorously followed Spanish FA mosaic population, have proposed diagnostic algorithms classifying individuals with fewer than 20% aberrant cells cultured in 0.1-μg/ml DEB as non-FA patients, 20–40% as consistent with FA mosaics, > 60% as consistent with FA, and 40–60% as possible mosaicism, with additional information regarding the number of breaks per cell further contributing to diagnosis [10]. Specific DNA-damaging agents, concentrations, other culture and cell selection conditions, and threshold aberrancy levels have varied among investigative centers such that no single standard for a determination of mosaicism exists. The presence of divergent DEB resistance between a patient’s lymphocytes and cultured fibroblasts is considered consistent with FA mosaicism, as is an increasing percentage of DEB-resistant lymphocytes over sequential intervals. A German-led international consortium of investigative centers has also identified mosaicism by decreased clastogen stimulation of G2-phase arrest [15]; French investigators have described normalized FANCD2 ubiquitination in the presence of clastogens as indicative of mosaicism [38]. As will be discussed subsequently, results of DEB-chromosomal stability evaluations in peripheral blood lymphocytes may not necessarily be indicative of reversions in other hematopoietic compartments, including multipotent HSPCs [14].

Gene sequencing has become an increasingly valuable and utilized modality for the confirmation of an FA diagnosis; however, sequencing of distinct blood and marrow progenitor cell populations has been performed infrequently and continues to be a predominantly research-focused modality [28, 37]. Evaluation of the MMC resistance of cultured bone marrow-derived colony forming cells (CFCs) has emerged as a complementary test for identifying phenotypic reversion in progenitor populations. The number of investigations in which this parameter has been correlated with long-term clinical outcomes is extremely limited [28, 37, 39]; however, the increasing percentage of MMC-resistant bone marrow CFCs has been identified in recent autologous gene therapy clinical trials and has been recognized as a relevant indicator of genetic and phenotypic reversal in pluripotent hematopoietic progenitor cells [36]. Ongoing discussions involving international multidisciplinary investigators have increasingly identified MMC resistance of bone marrow progenitors as meaningful and potentially objective clinical phenomena in either FA mosaicism or autologous hematopoietic gene therapy settings [40, 41]. Ongoing consensus discussions have also indicated the potential importance of evaluations employing multiple MMC concentrations, serial evaluations of MMC resistance over time, and corroboration of clastogen resistance with functional assays such as FANCD2 ubiquitination. When feasible, genetic sequencing and multilineage assessment are additionally relevant for comprehensive mosaicism assessment.

### Clinical outcomes in FA mosaic patients

In addition to the two illustrative and deeply investigated FA mosaic cases described previously, several additional series published over a 16-year period (1997–2013) have detailed the clinical course and essential laboratory correlates in FA mosaic patients. These include 4 publications and a published doctoral thesis, describing outcomes in FA mosaic patients (range: *n* = 5–14 per publication) [8, 14, 15, 38, 39]. Detailed information regarding these series is provided in Table 1. Essential themes emerging from these cohorts include the following:

1. FA mosaicism has largely been reported in cases arising from more frequently observed mutations in FA core and ubiquitination complex genes (predominantly *FANCA*, *FANCC*, and *FANCD2*); there is limited published information on FA mosaicism arising from mutations in downstream or more recently discovered FA genes/proteins [26, 42, 43].
2. Hematologic stability and improvement have been noted in a single lineage, or frequently in bi- and trilineage settings, consistent with the hypothesis that reversion mutations arise across a spectrum of pluripotent or more committed HSPCs [39].
3. A broad range of peripheral blood lymphocyte resistance to DEB (or other clastogens) has been reported, with a limited number of studies showing correlation between the percentages of resistant PB cells and clinical outcomes. (Of note, T lymphocyte mosaicism has been reported in up to 15–20% of FA patients and had been frequently associated with HSCT engraftment failure prior to the incorporation of fludarabine-based conditioning) [44].
4. FA mosaicism has been frequently but not universally associated with stable or increasing hematologic parameters over prolonged follow-up.
5. In the limited settings where normalization of blood counts has been observed over time, increases in blood lineages have been reported over a 1- to 6-year interval (most frequently over 2–3 years), and multilineage increases have been observed in non-contemporaneous (i.e., staggered) sequences as indicated in Table 1 [14, 38, 39].
6. In the limited settings where normalization of blood counts has been observed over time, platelet normalization has at times been the most indolent and incomplete relative to other lineages, with some increases resulting in levels below normal limits but not requiring clinical intervention, as indicated in Table 1 [14, 39].
7. Evaluation of MMC resistance in bone marrow CFCs has been undertaken infrequently. In the one series in which BM MMC resistance was evaluated, 5 of 5 patients with documented MMC resistance were alive and without hematologic complications at last follow-up (with stable clinical course over 9–15 years in 3 patients for whom this information was available) and 2 of 4 patients in whom marrow CFCs were sensitive to MMC died at ages 11 and 18 of BMF and unspecified complications [39].

**Table 1:** Fanconi Anemia Mosaicism: Publications Detailing Clinical Outcomes of Multi-patient Cohorts

Reference	Cohort (incl. Compln. Group)	Age & Duration of Follow-up	Clinical Observations	Laboratory Analyses & Clinical Correlates
Lo Ten Foe 1997	*n* = 8 FANCC *n* = 3 unknown *n* = 5	Age 9-30y *n* = 6 single eval. *n* = 2 f/u 22→28y & 3→16y	Mild hematologic deficits in 6 of 8 patients	▪ MMC-res. in 37-100% of PB lymphocytes ▪ Pt EUFA-192 followed age 3→16y with MMC-res. 0% (age 3y) → 95% (age 16y); clonal hematopoiesis demonstrated by PCR ▪ Gene conversion in 2 siblings (EUFA 449/50) with MMC-res 0% & 90% (respectively) & divergent hematologic course
Gross 2002	*n* = 5 FANCA *n* = 4 FANCC *n* = 1	Age 6-21y All followed over 3-6y interval	▪ Hematologic improvement in 4 of 5 patients ▪ PB increases in some pts noted over 3-6y period (platelet ↑ most delayed in several cases)	▪ Lymphoid-restricted reversion suspected in pt without hematologic improvement ▪ % of MMC-res PB lymphocytes not described (only breaks/cell)
Soulier 2005	*n* = 8 FANCA *n* = 5 FANCD2 *n* = 1 unknown *n* = 2	Age 4-34y Median f/u 5y (1-27 y)	▪ No BMF or AML/MDS in 8 mosaic patients (median f/u 5y) ▪ BMF/aplasia in 31 of 45 non-mosaic FA pts ▪ AML/MDS in 2 of 45 non-mosaic FA pts	▪ Normal PB lymphocyte FANCD2 ubiquitination pattern (immunoblot): 1° determinant of reversion ▪ Mechlorethamine 0.05μg/ml chromosomal breakage in PB lymphocytes: 20-56% (*n* = 4); negative (<20%; *n* = 3); ambiguous result in (*n* = 1)
	*n* = 45 non-mos. FA contr.	Age 2-36y (non-mos.)	▪ Normalization of Hb/Plt over 1&3 y w/ subsequent stabilization in 1 FANCA pt (27y f/u)	
Kalb 2007	*n* = 5 FANCD2	Age 9-34y (median 20y)	▪ Mild/protracted hematologic course in 3 pts (1 received androgens) although eventual BMF requiring Xfusn at age 17 & 18y in 2 pts ▪ Aggressive hematologic course in 2 pts (BMF age 4, 5) & death prior to age 10 despite HSCT (*n* = 1) or androgens (*n* = 2)	▪ Limited description of MMC-res of PBL/LCL ▪ 9-10% MMC-induced G2 phase arrest in in LCLs from *n* = 3 mosaic pts (8% in non-FA controls) ▪ Focus of publication was aggressive clinical course of FANCD2 & hypomorphic nature of many FANCD2 mutations
	*n* = 23 FANCD2 non-mos. contr.	F/u 0-18y (median 4y)		
Trujillo 2013	*n* = 34 FANCA *n* = 25 FANCD2 *n* = 3 FANCE *n* = 1 unknown *n* = 5	Detailed heme f/u for *n* = 14: over 3-22.5y (median 9.5-10y) Age 0-26.5y	▪ For *n* = 14 with detailed f/u: *n* = 6: tri-lineage normalization w/o BMF/AML age 14-26.5y (median 22-25y) *n* = 2: trilineage ↑ trend (age 12, 18y); plts < nl *n* = 3: bi-lineage normalization (↓plt *n* = 2) *n* = 3: death or BMF/HSCT (*n* = 1: no normalization; *n* = 2 some ↑/nl w/subsequent ↓↓) ▪ Normalization of decreased lineages over 2-6y, most frequently over 2-3 years	▪ *n* = 34 PB T-lymphocyte chromosomal fragility: 10-58% cells with aberrancies ▪ *n* = 9 w/ evaluation of BM MMC-resistance: 5 of 5 pts w/MMC-res alive at last f/u 2 of 4 pts w/ MMC-sensitivity died at ages 11, 18y

An additional investigation based on these and other published FA mosaic cases is described in a subsequent section of this review. We undertook this literature-based investigation with an intent of identifying a subset of FA mosaic patients most likely to harbor reversion mutations in long-term repopulating HSCs.

### Hypomorphic mutations in FA

Relatively little has been reported regarding the incidence or clinical significance of hypomorphic mutations in FA, in which the impacted protein is expressed with reduced quantity and/or function. Hypomorphic mutations have been described in FANCD2 and were not associated with any reduction in the typically aggressive hematologic manifestations associated with this complementation group; the incidence of mosaicism in FANCD2 also appears comparable to that in FANCA or other core complex FA groups [15].

Recently, a cohort of 11 Sicilian patients with *FANCA* hypomorphic mutations were reported, in which mutations resulted in FANCA proteins that localized to cytoplasm and facilitated more normalized mitochondrial function (relative to cells lacking FANCA), with peripheral blood-derived lymphoblast sensitivity comparable to non-mosaic FA patients. The clinical course of this cohort ranged from indolent (*n* = 3) to severe (*n* = 2), with the majority of patients (*n* = 6) encountering moderate single-, bi-, or trilineage cytopenias and receiving allogeneic hematopoietic transplant at ages 9–21 [45]. It is likely that as more comprehensive genetic sequencing becomes clinically feasible, additional hypomorphic mutations may be recognized, including some with potential for more indolent clinical progression resemblant of mosaicism, but without the clastogen resistance associated with compensatory mutations in FA. Additional investigations into the mitochondrial and other “non-canonical” functions of FA proteins may also elucidate potential prognoses associated with these uncommon variants [46].

## Review of FA mosaic publications with emphasis on blood count normalization

### Overview and objectives

The clinical significance of FA mosaicism remains uncertain more than 20 years after this phenomenon was initially reported. We sought a comprehensive literature-based understanding of FA mosaicism with emphasis on cases most likely emanating from reversion mutations in long-term repopulating HSC populations. Focusing on the minority of FA mosaic cases in which normalization of all peripheral blood lineages was reported, we identified a population of FA mosaic patients in whom there appears to be high likelihood of multi-decade BMF-free and leukemia-free survival.

Our objectives were to identify FA mosaicism cases reported in the medical literature for which there was information regarding individual patient clinical outcomes and to further identify the subset of FA mosaic cases in which there was normalization of all peripheral blood lineages (because such cases likely result from reversion mutations in long-term repopulating HSC populations) and to determine clinical course in this clinical subpopulation. In particular, we sought to quantify the incidences of BMF, hematologic malignancy, and other adverse outcomes in FA mosaic patients, including those with blood count normalization and those with no or incomplete normalization.

### Methods

We identified published FA mosaic cases via PubMed searches, reference citations, and a publicly available doctoral thesis on this specific condition. A database was constructed from cases in which there was sufficient clinical information enabling evaluation of age at FA and mosaic diagnosis, duration of follow-up and development of BMF, malignancy, allogeneic hematopoietic stem cell transplant (HSCT), survival, and cause of death. Blood count normalization was defined as improvement in absolute neutrophil count (ANC) to at least 1000/mm^3^, hemoglobin to at least 9 g/dL, and platelets to at least 50,000/mm^3^ after previously lower levels or maintenance of these levels over multiple intervals in settings where earlier counts were not available or presence of these levels at a single timepoint with prior/subsequent hematologic stability reported by the investigator or blood counts normalized or within normal range per investigator without precise values specified.

### Results

**Fig. 2:**
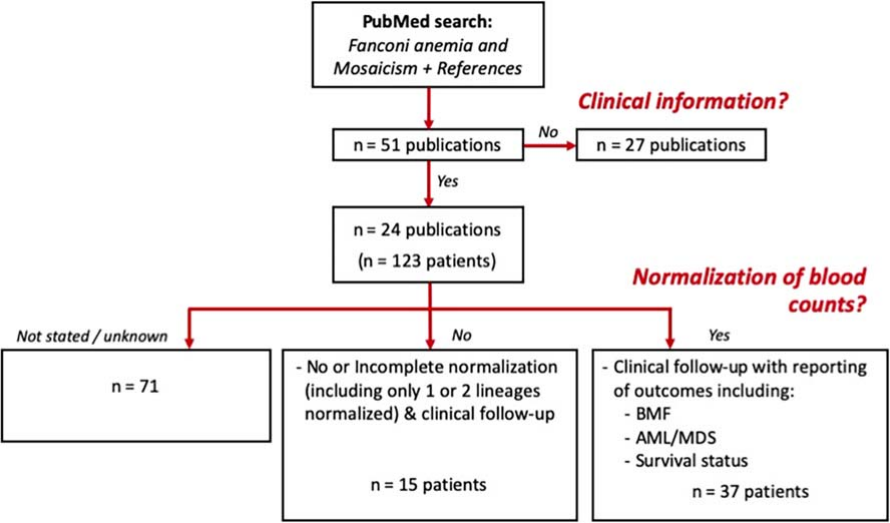
In the cohort of FA mosaic patients (*n* = 37) with normalized blood counts and information regarding clinical outcomes, individuals were included from the following cohorts: Spain (*n* = 13), Germany (*n* = 10), France (*n* = 8), the USA (*n* = 5), and Japan (*n* = 1). Complementation groups of patients identified in this cohort included: Group A (*n* = 24), Group B (*n* = 1), Group C (*n* = 1), Group E (*n* = 1), and Group T (*n* = 1); in *n* = 7 patients, complementation group was unknown

Figure 2 details the literature search results in which 51 publications describing FA mosaicism were identified between 1976 and 2018, of which 23 publications (*n* = 123 patients) contained clinical information. A subset of 37 patients (30%) had normalization of blood counts (including 19 for whom detailed blood count information over multiple timepoints was reported). Fifteen FA mosaic patients were reported in whom no or incomplete normalization (including normalization of 1 or 2 lineages) was described. Blood count information was not reported in 71 patients. The subgroup of 37 FA mosaic patients with normalized blood counts were reported from investigators in Spain (*n* = 13), Germany (*n* = 10), France (*n* = 8), the USA (*n* = 5), and Japan (*n* = 1). Complementation groups of patients in this cohort were Group A (*n* = 24), Group B (*n* = 1), Group C (*n* = 1), Group E (*n* = 1), Group T (*n* = 1), and not reported/known (*n* = 9). Publications detailing these patients are shown in Supplemental Table 1. As indicated previously, there is limited published information on FA mosaicism arising from mutations in downstream or more recently discovered FA genes/proteins.

**Fig. 3:**
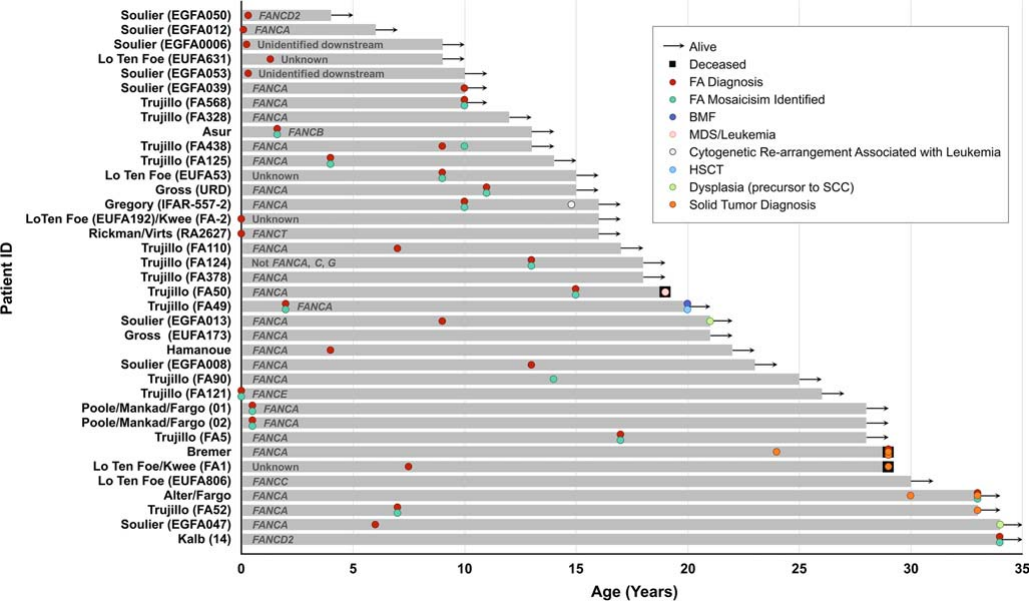
Swimmer plot depicting clinical course for 37 FA mosaic patients with normalized blood counts, including age at diagnosis (when available), last follow-up, and occurrences of BMF, AML/MDS, allogeneic HSCT, and solid organ cancers. The majority of patients (34 of 37) were alive without BMF, malignancy, or transplant at last follow-up. Criteria for normalization are provided in the text and are in general more stringent (exclusive) than those applied by investigators in Table 1

The clinical outcomes for the 37 FA mosaic patients with normalized blood counts are depicted in Fig. 3. Median follow-up within this subgroup was to age 18 (range 4–34 years). Thirty-four patients (92%) were alive at last follow-up. Death in 3 patients occurred at ages 19 (AML), 29 (lung cancer), and 29 (head and neck cancer). BMF was reported in one patient (3%, age 20) and leukemia (AML/MDS) in one patient (3%, age 19). An additional patient developed an 11q deletion (MLL rearrangement) in a clonal population persistent over 2 years of follow-up but without overt evidence of leukemia. Solid organ malignancies were reported in 4 patients (11%, ages 24–33) including 2 patients with multiple sequential cancer diagnoses. Additional details regarding these outcomes are provided in Table 2. The clinical outcomes for the 15 FA mosaic patients in whom no or incomplete normalization was reported were more adverse and are provided in Table 2; 8 of these patients (53%) were alive at last follow-up with 7 deaths occurring between ages 9 and 39. BMF was reported in 12 of 15 patients (80%, median age 9.5 years); 2 of 15 patients (13%) developed AML/MDS, and 2 (13%) developed solid organ malignancies.

**Table 2:** Clinical outcomes for Fanconi anemia mosaic patients

A. Mosaic with blood count normalization (*n* = 37)			
Status	*n* (%)	Age (y)	
Alive at last follow-up	34 (92%)	At last follow-up:	Median: 18 Range: 4–34
Deceased at follow-up	3 (9%)	At death:	19, 29, 29
BMF	1 (3%)	At BMF diagnosis:	20
AML/MDS	1 (3%)	At AML/MDS diagnosis:	19
Solid organ malignancy	4 (11%)	At cancer diagnosis:	Median: 29 Range: 24–33
B. Mosaic with no/incomplete blood count normalization (*n* = 15)			
Status	*n* (%)	Age (y)	
Alive at last follow-up	8 (53%)	At last follow-up:	Median:14 Range: 8–47.5
Deceased at follow-up	7 (47%)	At death:	Range: 9–39
BMF	12 (80%)	At BMF diagnosis:	Median: 9.5 Range: 0–47.5
AML/MDS	2 (13%)	At AML/MDS diagnosis:	17.5, 47.5
Solid organ malignancy	2 (13%)	At cancer diagnosis:	38, 39

FA mosaic patients with blood count normalization were less likely to develop BMF (*p* < 0.0001, Fisher’s Exact Test, 2-sided) and more likely to remain alive (*p* = 0.0034) relative to FA mosaics without normalization. A lower percentage of mosaic patients with normalization developed hematologic malignancy (3% vs. 14%), but this difference was not statistically significant. The incidences of solid organ malignancies were similar between these subgroups.

Based on these findings, FA mosaicism with normalized blood counts appears to occur in a minority (30%) of patients whose peripheral lymphocyte assessments indicate resistance to DNA-damaging agents via standard testing; the potential for reporting bias is such that the actual incidence may be lower; it is likely that fewer than 5% of all FA patients experience blood count normalization as a result of reversion/compensatory mutations. FA mosaicism with normalized blood counts is associated with very limited incidences of BMF or hematologic malignancy, limited requirement for allogeneic HSCT, and relatively limited mortality during the initial 2–4 decades of life. Solid tumors arise during the 3rd and 4th decades of life in FA mosaic patients regardless of blood count normalization, as is the case with non-mosaic FA patients. Two of four FA patients with normalized blood counts who developed solid tumors had a second malignancy diagnosed within several years of the initial cancer diagnosis; solid tumors were fatal in at least 2 of 4 reported patients.

### Discussions and summary

These findings indicate that there is a population of FA mosaic patients with very limited risk of BMF or hematologic malignancy, likely as a result of reversion mutations in primitive HSPC populations. Limitations for conclusive analysis result from the absence of bone marrow-based genetic or mitomycin-resistance evaluation in the overwhelming majority of FA mosaic cases. Analysis is also limited because sequential chronologic assessment of peripheral blood or bone marrow clastogen resistance has been performed infrequently. It is very likely that FA mosaicism with normalized blood counts is the result of mutations in long-term HSPCs, but it is not known what percentage of HSPC with reversion events is required to enable stable or normalized peripheral blood cell counts [28, 37]. Presumably those mosaic patients in whom no or incomplete normalization occurred were diagnosed as a result of reversions in committed progenitor (lymphoid-inclusive) lineage or short-term repopulating progenitor cells, but more detailed genetic evaluation of progenitor lineages will be needed to validate this hypothesis. Additional nongenetic elements (including epigenetic factors, stromal microenvironment, telomeric depletion, and aldehyde exposure and metabolism) may also contribute to hematopoietic stability in settings of genetic correction [5, 47, 48]. It is plausible that reversion events may only result in favorable clinical outcomes when these take place in long-term repopulating HSCs and additional favorable metabolic or other nongenetic conditions. A case of mosaicism emerging subsequent to initiation of androgen therapy has been reported, with blood count normalization persisting subsequent to androgen discontinuation (duration undetermined); it is plausible that androgens may selectively enhance hematopoiesis in dormant revertant HSCs and that some cases of prolonged androgen-attributed hematologic benefit were in fact the result of undetected mosaicism [49]. Prospective translational studies will be required to enable optimal understanding of genotype/phenotype correlations in settings of reversion mutations or therapeutic genetic correction – these are planned in the context of the next generation of gene therapy clinical investigations.

Clinical trials of autologous gene therapy in FA have been conducted during the prior decades, with most recent results indicating genetic correction and reversal of sensitivity to DNA-damaging agents at levels resembling those seen in FA mosaic patients [35, 50, 51]. Our findings also provide rationale for further clinical evaluation of gene therapy because they indicate that there is a likely threshold of genetic and phenotypic correction above which multi-decade BMF-free survival may be likely. These comparisons between FA mosaic and gene therapy treated patients are limited in that mosaicism likely results from a single-cell mutation (or mutation within a very finite subset of cells), whereas gene therapy involves introduction of a likely higher number of gene-corrected HSPCs with oligoclonal hematopoietic repopulation observed to date [36]. The long-term significance of the oligoclonal hematopoiesis identified in nonconditioned gene therapy recipients (and suspected in FA mosaic patients) is uncertain, although there exist similarities to the clonal hematopoiesis that has become increasingly recognized as a distinct phenomenon, particularly in elderly individuals. Clonal hematopoiesis in the elderly occurs frequently and, even in the absence of mutations identified with hematologic malignancy, is associated with increased risk of hematologic malignancy and cardiovascular events, although the absolute risk in individual patients is relatively limited [52, 53]. Long-term follow-up studies of the initial gene therapy treated FA patients are underway, although multi-year observation will likely be required to enable definitive conclusions regarding the ramifications of oligoclonal hematopoiesis.

In summary, FA mosaicism is a condition in which varied and frequently divergent degrees of resistance to DNA damage are present within the hematopoietic system of an individual patient, or (more rarely) the entire hematopoietic system displays evidence of normal or near-normal resistance to DNA damage despite clastogen sensitivity in non-hematopoietic tissues. These findings are at times accompanied by normalized or stable peripheral blood counts, with some patients surviving into adulthood without bone marrow failure or hematologic malignancy. Some degree of mosaicism is likely present in 15–20% of FA patients, although the incidence of reversion mutations in primitive HSCs resulting in sustained hematologic stability likely occurs in fewer than 5% [9, 10, 15, 38]. Our additional evaluation of 37 cases with documented blood cell count normalization suggests that there is a population of FA patients in whom a sufficient genetic reversion and selective advantage enable hematologic stability through at least three decades with limited BMF or myeloid malignancy. The long-term results of gene therapy studies – especially those involving no conditioning – are likely to provide important information as to the clinical relevance of reversion mutations and other forms of genetic correction in this complex disorder.

## Electronic supplementary material

ESM 1 (DOCX 21.9 kb)
