# Supplementary material for: Mosaicism in Fanconi anemia: concise review and evaluation of published cases with focus on clinical course of blood count normalization
Source: Ann Hematol. 2020 Feb 17;99(5):913–24. doi: 10.1007/s00277-020-03954-2 (PMC7196946; doi:10.1007/s00277-020-03954-2)
Supplement: Supplementary file 1 — (DOCX 21.9 kb) [file 277_2020_3954_MOESM1_ESM.docx]

**Supplemental Table 1 Publications Describing Clinical Outcomes of FA Mosaic Patients (including those with and without blood count normalization)**

| **Reference** | **# of Mosaic Patients in Report** | **# of Mosaic Patients with Blood Count Normalization** |
| --- | --- | --- |
| Trujillo Quintero JP (2013) Genética clínica la anemia de Fanconi. Dissertation, Universitat Autónoma de Barcelona. Doctoral thesis; Autonomous University of Barcelona. <https://ddd.uab.cat/pub/tesis/2013/hdl_10803_129106/jptq1de1.pdf> [39] | 14 | 13 |
| Lo Ten Foe JR, Kwee ML, Rooimans MA, Oostra AB, Veerman AJ, van Weel M, et al (1997) Somatic mosaicism in Fanconi anemia: molecular basis and clinical significance. Eur J Hum Genet 5(3):137-148 [8] | 8 | 5 |
| Soulier J, Leblanc T, Largher J, Dastot H, Shimamura A, Guardiola P, et al (2005) Detection of somatic mosaicism and classification of Fanconi anemia patients by analysis of the FA/BRCA pathway. Blood 105:1329-1336 [38] | 8 | 8 |
| Gross M, Hanenberg H, Lobitz S, Friedl R, Herterich S, Dietrich R et al (2002) Reverse mosaicism in Fanconi anemia: natural gene therapy via molecular self-correction. Cytogenet Genome Res 98(2-3):126-135 [14] | 5 | 2 |
| Kalb R, Neveling K, Hoehn H, Schneider H, Linka Y, Batish SD, et al (2007) Hypomorphic mutations in the gene encoding a key Fanconi anemia protein, FANCD2, sustain a significant group of FA-D2 patients with severe phenotype. Am J Hum Genetics 80:895-910 [15] | 5 | 1 |
| Pinto F, Leblanc T, Chamousset D, Le Roux G, Brethon B, Cassinat B, et al (2009) Diagnosis of Fanconi anemia in patients with bone marrow failure. Haematologica 94(4):487-495 | 3 | 0 |
| Poole SR, Smith AC, Hays T, McGavran L, Auerbach AD (1992) Monozygotic twin girls with congenital malformations resembling Fanconi anemia. Am J Med Genet 42:780-784 [29]  Mankad A, Taniguchi T, Cox B, Akkari Y, Rathbun RK, Lucas L, et al (2006) Natural gene therapy in monozygotic twins with Fanconi anemia. Blood 107:3084-3090 [28]  Fargo JH, Rochowski A, Giri N, Savage SA, Olson SB, Alter BP (2014) Comparison of chromosome breakage in non-mosaic and mosaic patients with Fanconi anemia, relatives, and patients with other inherited bone marrow failure syndromes. Cytogenet Genome Res 144(1):15-27 [9] | 2^b^ | 2^b^ |
| Dokal I, Chase A, Morgan NV, Coulthard S, Hall G, Mathew CG, et al (1996) Positive diepoxybutane test in only one of two brothers found to be compound heterozygotes for Fanconi’s anemia complementation group C mutations. Br J Haematol 93:813-816 | 1 | 0 |
| Gregory JJ, Jr, Wagner JE, Verlander PC, Levran O, Batish SD, Eide CR et al (2001) Somatic mosaicism in Fanconi anemia: evidence of genotypic reversion in lymphohematopoietic stem cells. Proc Natl Acad Sci USA 98:2532-2537 [37] | 1 | 1 |
| Bremer M, Schindler D, Groβ M, Dörk T, Morlott S, Karstens JH (2003) Fanconi’s anemia and clinical radiosensitivity. Strahlenther Onkol 11:748-753 | 1 | 1 |
| Alter BP, Joenje H, Oostra AB, Pals G (2005) Adult head and neck cancer and hematopoietic mosaicism. Arch Otolaryngol Head Neck Surg 131:635-639  Fargo JH, Rochowski A, Giri N, Savage SA, Olson SB, Alter BP (2014) Comparison of chromosome breakage in non-mosaic and mosaic patients with Fanconi anemia, relatives, and patients with other inherited bone marrow failure syndromes. Cytogenet Genome Res 144(1):15-27 [9] | 1^c^ | 1^c^ |
| Hamanoue S, Yagasaki H, Tsuruta T, Oda T, Yabe H, Yabe M, et al (2005) Myeloid lineage-selective growth of revertant cells in Fanconi anemia. Br J Haematol 132:630-636 | 1 | 1 |
| Rickman KA, Lach FP, Abhyankar A, Donovan FX, Sanborn EM, Kennedy JA, et al (2015) Deficiency of UBE2T, the E2 ubiquitin ligase necessary for FANCD2 and FANCI ubiquitination, causes FA-T subtype of Fanconi anemia. Cell Rep 12(1):35-41 [42]  Virts EL, Jankowska A, Mackay C, Glaas MF, Wiek C, Kelich SL, et al (2015) AluY-mediated germline deletion, duplication and somatic stem cell reversion in UBE2T defines a new subtype of Fanconi anemia. Hum Mol Genet 24(18): 5093-5108 [43] | 1^a^ | 1^a^ |
| Asur RS, Kimble DC, Lach FP, Jung M, Donovan FX, Kamat A et al (2018) Somatic mosaicism of an intragenic FANCB duplication in both fibroblast and peripheral blood cells observed in a Fanconi anemia patient leads to milder phenotype. Mol Genet Genomic Med 6:77-91 [26] | 1 | 1 |

Fourteen publications are listed in order (descending) of numbers of patients described, including n=52 patients overall (n=37 with blood count normalization)
a. Patient is described in both Rickman (2015) and Virts (2015) publications

b. Patients are described in Poole (1992), Mankad (2006), and Fargo (2014) publications

c. Patient is described in Alter (2005) and Fargo (2014) publications
